# Comparative physiological and transcriptomic analyses of photosynthesis in *Sphagneticola calendulacea* (L.) Pruski and *Sphagneticola trilobata* (L.) Pruski

**DOI:** 10.1038/s41598-020-74289-1

**Published:** 2020-10-20

**Authors:** Min-Ling Cai, Qi-Lei Zhang, Jun-Jie Zhang, Wen-Qiao Ding, Hong-Ying Huang, Chang-Lian Peng

**Affiliations:** 1 Guangzhou Key Laboratory of Subtropical Biodiversity and Biomonitoring, Guangdong Provincial Key Laboratory of Biotechnology for Plant Development, College of Life Sciences, South China Normal University, Guangzhou, 510631 People’s Republic of China; 2 College of Chemistry & Biology and Environmental Engineering, Xiangnan University, Chenzhou, 423043 Hunan People’s Republic of China

**Keywords:** Ecology, Genetics, Physiology, Plant sciences

## Abstract

*Sphagneticola trilobata* (L.) Pruski is one of the fast-growing malignant weeds in South China. It has severely influenced local biodiversity and native plant habitat. Photosynthesis is the material basis of plant growth and development. However, there are few reports on the photosynthetic transcriptome of *S. trilobata*. In this study, *S. trilobata* had a relatively large leaf area and biomass. The gas exchange parameters per unit area of leaves, including net photosynthetic capacity (P_n_), intercellular CO_2_ (C_i_), stomatal conductance (G_s_), transpiration rate (T_r_), water use efficiency (WUE), photosynthetic pigment and Rubisco protein content were higher than those of the native plant *Sphagneticola calendulacea* (L.) Pruski. On this basis, the differences in photosynthesis pathways between the two *Sphagneticola* species were analyzed by using the Illumina HiSeq platform. The sequencing results for *S. trilobata* and *S. calendulacea* revealed 159,366 and 177,069 unigenes, respectively. Functional annotation revealed 119,350 and 150,846 non-redundant protein database annotations (Nr), 96,637 and 115,711 Swiss-Prot annotations, 49,159 and 60,116 Kyoto Encyclopedia of Genes and Genomes annotations (KEGG), and 83,712 and 97,957 Gene Ontology annotations (GO) in *S. trilobata* and *S. calendulacea*, respectively. Additionally, our analysis showed that the expression of key protease genes involved in the photosynthesis pathway, particularly *CP43*, *CP47*, *PsbA* and *PetC*, had high expression levels in leaves of *S. trilobata* in comparison to native species. Physiological and transcriptomic analyses suggest the high expression of photosynthetic genes ensures the high photosynthetic capacity of leaves, which is one of the inherent advantages underlying the successful invasion by *S. trilobata*.

## Introduction

When a species is introduced into a new environment, it may not adapt to the new environment, it may die out quickly, or it may resist the external environment to survive and reproduce. The species that survive may become competitive and settle in new areas and niches at high speed, eventually becoming successful invaders. In the twenty-first century, the biological invasion is one of the main environmental threats to biodiversity worldwide. It not only destroys the biodiversity, structure and function of the ecosystem, but also causes substantial economic losses to the invaded areas^1–3^. China has become one of the countries that is most severely invaded by foreign organisms and has the highest proportion of exotic plants worldwide^4^.

*Sphagneticola trilobata* (L.) Pruski (Asteraceae), a creeping perennial herb, is native to South America. In the 1970s, *S. trilobata* was introduced into China as a groundcover plant and quickly escaped into the wild. Now it has become the most common weed in southern China, crowding out native plant *Sphagneticola calendulacea* (L.) Pruski and forming a single excellent community^5,6^. *S. trilobata* has been recognized as one of the 100 most harmful invasive species in the world^7^. At present, there is a broad understanding of the invasion biology of exotic plants, indicating that the factors affecting plant invasion mainly include the invasiveness of communities of exotic plants and their native congeners^8^. Various factors in an ecosystem may affect the productivity of native and invasive species, including changes in the external environment^9^, the inherent biodiversity^10^, and competitive intra- and interspecies relationships^11^, such as rapid reproduction capacity, extensive ecological adaptability and strong allelopathy. However, research on these characteristics has mainly focused on leaves^12–14^. As the most important organs for photosynthesis, leaves are sensitive to changes in the external environment during evolution^15^. Previous studies have shown that the leaves of *S. trilobata* have a higher CO_2_ fixation capacity, wider photosynthetic effective radiation range and higher light quantum utilization efficiency than *S. calendulacea*^16,17^. The construction cost of its leaves is significantly lower than that of *S. calendulacea*, but its distribution is easily limited by water^18^. These results indicate that photosynthesis is of great significance in the successful invasion of *S. trilobata*. However, research on the invasion mechanisms of *S. trilobata* mainly focuses on the physiological and ecological aspects of leaves, while research at the photosynthetic transcriptional level is rarely reported^16,18^.

The comprehensive expression profiles of genes involved in photosynthesis in *S. trilobata* are important for understanding the molecular basis and the differences in gene expression compared to that in *S. calendulacea*. Development of the Illumina next-generation sequencing technique has provides a convenient and effective means to obtain genome resources from non-model species^19–21^. The technique also allows the effective determination of the genetic basis and mechanism of successful invasion based on a comparative study of invasive and noninvasive species, including *Ipomoea cairica*^22^, *Spartina alterniflora*^23^, and *Alternanthera philoxeroides*^24^. In this study, we used the method of de novo sequencing for assembly and annotation. Using a combination of photosynthetic physiology and transcriptomic data, we analyzed the differences in photosynthesis between *S. trilobata* and *S. calendulacea*. Our study provides a material basis for comprehensively elucidating the invasion mechanism of *S. trilobata* at the transcriptome level in the future.

## Results

### Photosynthetic physiological characteristics of leaves of two species

#### Comparison of phenotype and biomass in leaves

**Figure 1:**
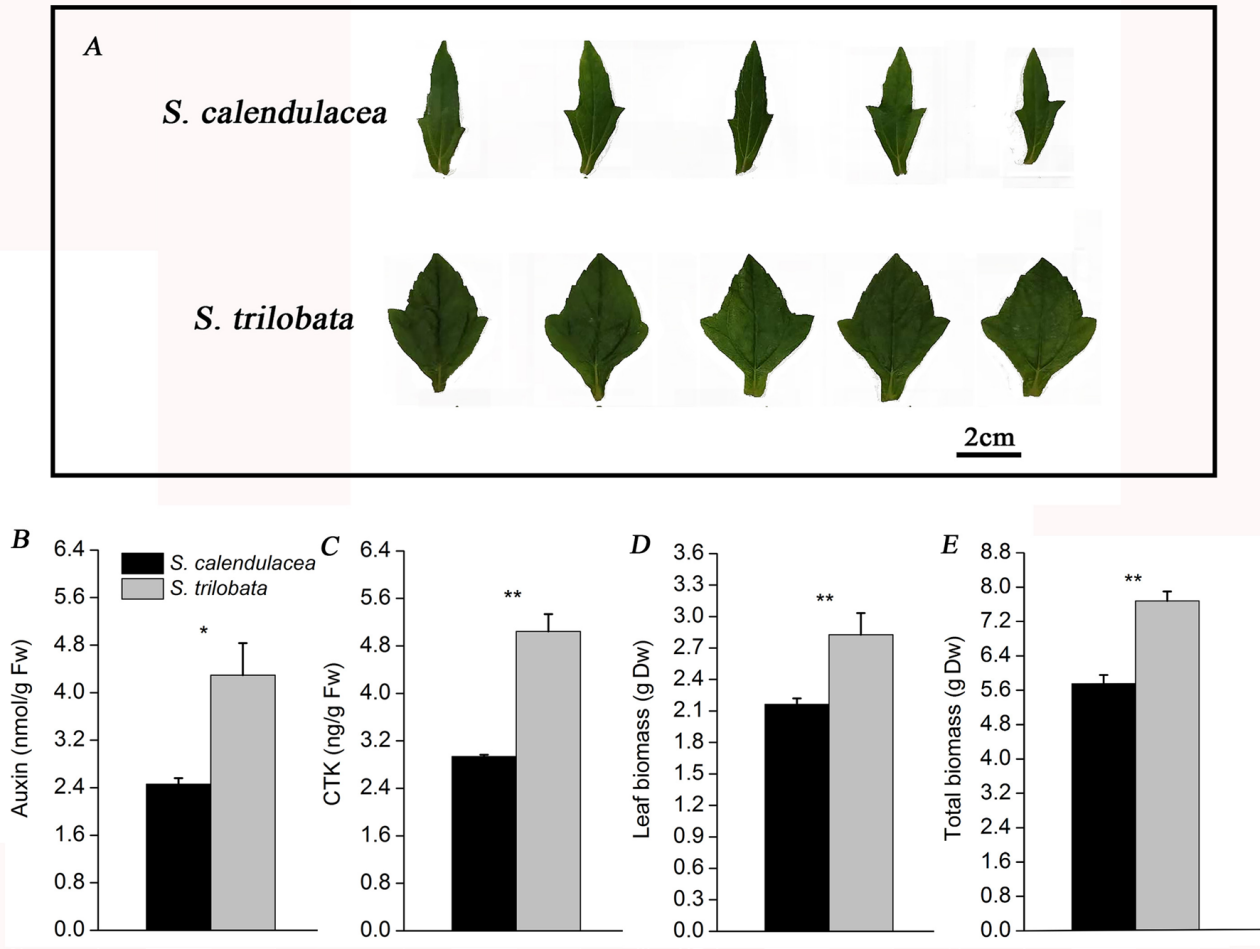
Phenotypes of *S. calendulacea* and *S. trilobata* (**A**)*.* Changes in plant hormone content, including auxin (**B**), and cytokinin, CTK (**C**). Difference in biomass between *S. calendulacea* and *S. trilobata,* leaf biomass (**D**) and total biomass (**E**). The error bars represent the standard deviations (SDs) of five to eight biological replicates, and the asterisks indicate significant differences (two-sided Student’s t-test, *P < 0.05, **P < 0.01).

With regard to the phenotypes of the two species, the leaves of *S. trilobata* were wide and large, nearly twice as large as those of *S. calendulacea* (Fig. 1A). The results showed that the plant hormone content including the auxin and cytokinin (CTK) content, in the leaves of *S. trilobata* was nearly twice as high as that in the native species, which may be one of the reasons for ensuring the larger leaf area of an invasive plant (Fig. 1B,C). The leaf and total biomass of *S. trilobata* were higher than those of *S. calendulacea* (Fig. 1D,E). These phenomena indicated that the growth of *S. trilobata* was better than that of *S. calendulacea* in the invaded area.

#### Comparison of photosynthetic capacity in leaves

**Figure 2:**
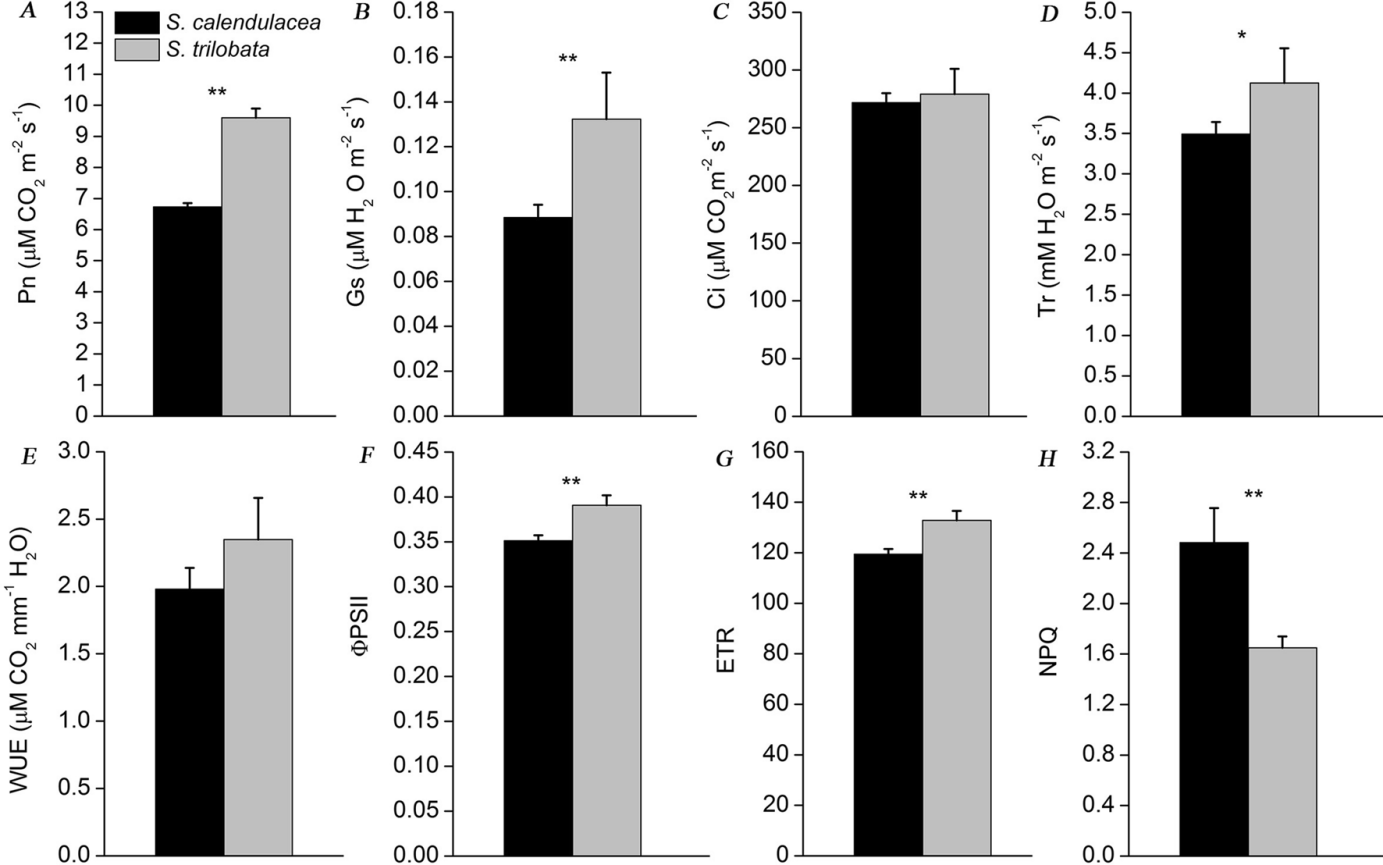
Gas exchange parameters of *S. trilobata* and *S. calendulacea,* including the net photosynthetic rate (P_n_) (**A**), stomatal conductance (G_s_) (**B**), and intercellular CO_2_ concentration (C_i_) (**C**), transpiration rate (T_r_) (**D**), and water use efficiency (WUE) (**E**) (n = 5). Chlorophyll fluorescence parameters including the electron transfer rate (ETR) (**F**) and actual photochemical efficiency (Φ_PSII_) in the leaves of the two species (**G**). The error bars represent the standard deviations (SDs) of five biological replicates, and the asterisks indicate significant differences (two-sided Student’s t-test, *P < 0.05, **P < 0.01).

**Figure 3:**
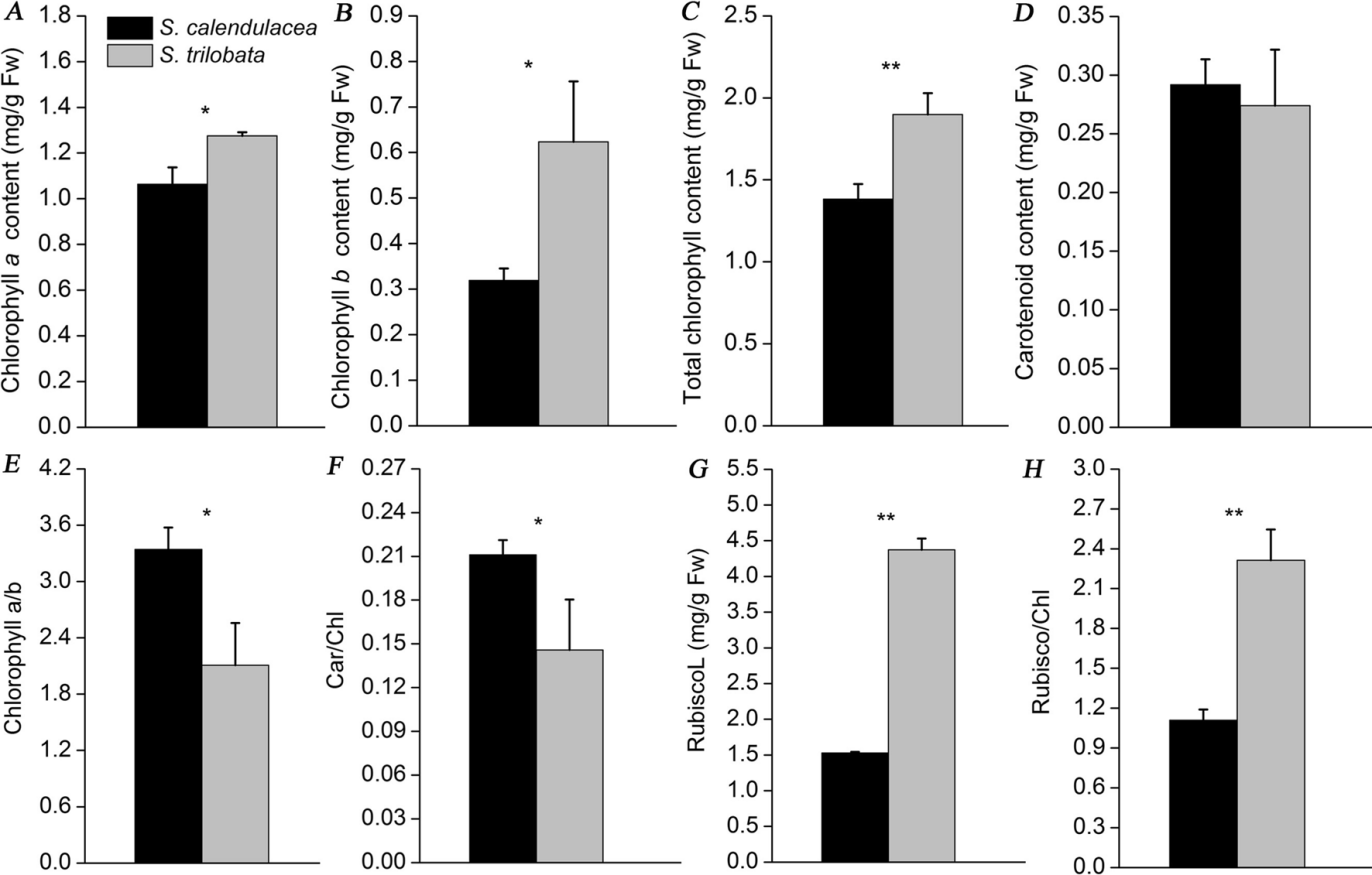
Contents of photosynthetic pigments and protein in *S. calendulacea* and *S. trilobata* (n = 5)*.* Contents of chlorophyll *a* (Chl *a*) (**A**), chlorophyll *b* (Chl *b*) (**B**), total chlorophyll (Chl) (**C**), carotenoid (Car) (**D**), the ratio of Chl *a/b* (**E**) and Car/Chl (**F**). Rubisco content (**G**) and the value of Rubisco/Chl (**H**) in the two species. The error bars represent the standard deviations (SDs), and the asterisks indicate significant differences according to two-sided Student’s t-test (*P < 0.05, **P < 0.01).

Photosynthesis is an important material basis for plant growth and development^25^. In this study, we compared the differences in photosynthetic capacity between two species. The results showed that the net photosynthetic rate (P_n_) of *S. trilobata* was significantly higher than that of its native congener *S. calendulacea* (Fig. 2A). The intercellular CO_2_ concentration (C_i_) and transpiration (T_r_) had the same trend as P_n_ while the stomatal conductance (G_s_) and water use efficiency (WUE) were slightly higher than those of *S. calendulacea* (Fig. 2B–E). The photosynthetic rate of a plant is closely related to the electron transfer in chloroplasts. Compared with *S. calendulacea*, the chlorophyll (Chl) fluorescence parameters showed that the leaves of *S. trilobata* had higher electron transfer ability, for which the electron transport rate (ETR) value was close to 130 and the actual photochemical efficiency (Φ_PSII_) had the same trend as ETR (Fig. 2F,G). However, the non-photochemical quenching (NPQ) was significantly higher in *S. calendulacea* than *S. trilobata* (Fig. 2H)*.* These results indicated that PSII of *S. trilobata* had high activity and strong photosynthetic capacity. Besides, the contents of photosynthetic pigment and Rubisco protein in leaves were also important indicators of photosynthetic capacity. The contents of Chl*a*, Chl*b* and Chl were also high in the leaves of *S. trilobata* while Carotenoid (Car) content in *S. calendulacea* was slightly higher than that in invasive plants (Fig. 3A–D). In contrast, the Chl*a*/*b* and Car/Chl ratios in *S. trilobata* were lower than those in *S. calendulacea* (Fig. 3E,F). Moreover, *S. trilobata* also had higher (by nearly three-fold) Rubisco content in the leaves than *S. calendulacea* (Fig. 3G). The Rubisco/Chl ratio had the same trend as Rubisco (Fig. 3H).

### Transcriptome sequencing analysis of leaves of two species

#### Sequencing and de novo assembly

Total RNA from the leaves of the two species was extracted and sent to the Novogene for construction of a cDNA library, which was sequenced using the Illumina HiSeq platform. After de novo assembly and removal of low-quality reads, 470,401 contigs with an average length of 762.06 bp were obtained for *S. calendulacea*, compared with 429,536 contigs with a length of 762.92 bp for *S. trilobata*. Sequence clustering was performed by CD-HIT, and the total numbers of *S. calendulacea* and *S. trilobata* unigenes were 177,069 and 159,366, with average lengths of 645.96 bp and 660.05 bp, respectively. The quality evaluation of the unigenes showed that the GC content and N50 values of *S. calendulacea* and *S. trilobata* were 39.18%/1025 bp and 38.76%/1037 bp, respectively (Table 1).

**Table 1:** Summary of de novo sequence assembly in *S. calendulacea* and *S. trilobata.*

	*S. calendulacea*	*S. trilobata*
Total number of contigs	470,401	429,536
Means length of contigs (bp)	762.06	762.92
Total number of unigenes	177,069	159,366
Means length of unigenes (bp)	645.96	660.05
Percent GC	39.18	38.76
N50	1025	1037

**Figure 4:**
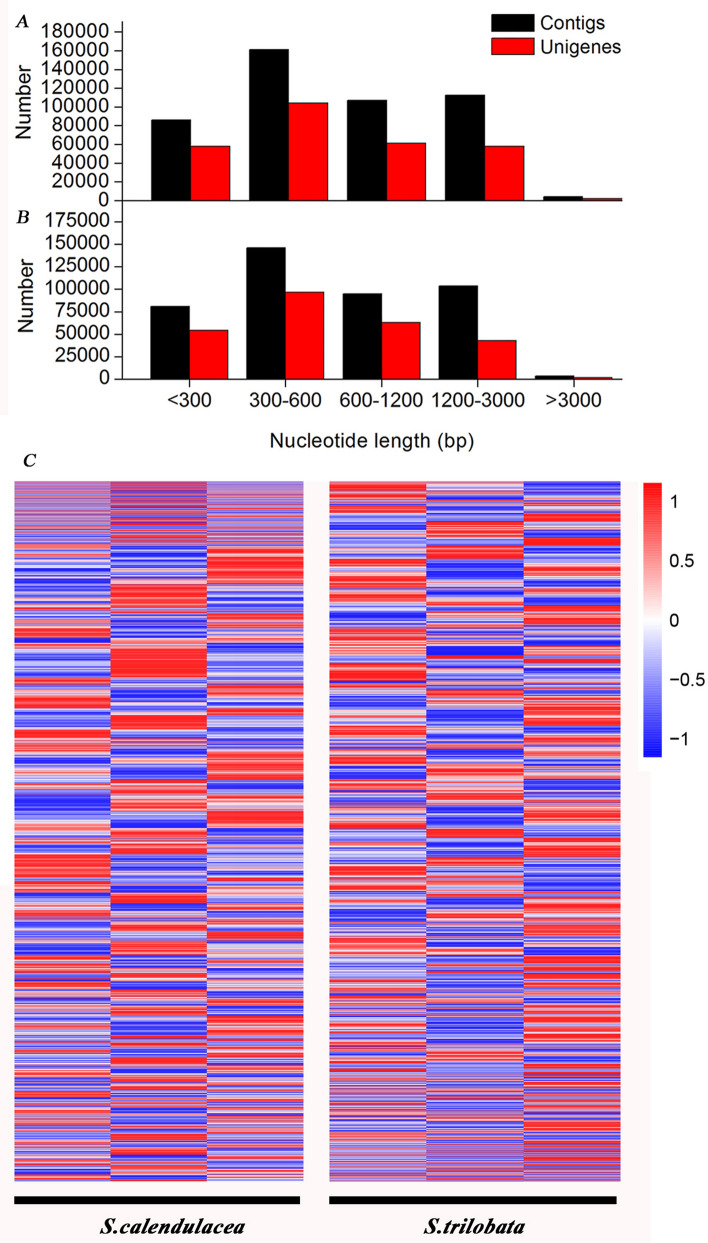
The numbers of contigs and unigenes and heatmaps of gene expression. Comparison of the numbers of contigs and unigenes in two species, *S. calendulacea* (**A**) *and S. trilobata* (**B**)*.* Black and red bars indicate contigs and unigenes, respectively. The x-axis on the bottom shows the number of contigs and unigenes classified in each sizes range. The y-axis indicates the number of genes. Heatmap of gene expression in *S. calendulacea* (left) *and S. trilobata* (right) (**C**)*.*

In *S. calendulacea*, the number of contigs with sequence lengths was less than 300 bp was 86,091 (18%). A total of 161,137 (34%) contigs ranged in length from 300–600 bp with a high number of genes. The number of contigs ranging in length from 600–1200 bp and the number of contigs ranging in length from 1200–3000 bp was similar (106,811 (23%) and 112,378 (24%), respectively), and the remaining contigs were longer than 3000 bp (Fig. 4A). In *S. trilobata*, the length of approximately 80,854 (19%) contigs was less than 300 bp. A total of 146,284 (34%) contigs ranged in length from 300–600 bp. The number of contigs ranging in length from 600–1200 bp and 1200–3000 bp was similar (95,103 (22%) and 103,523 (24%), respectively), and the remaining contig lengths were longer than 3000 bp (Fig. 4B). However, heatmap analysis showed the differences in gene expression between *S. calendulacea and S. trilobata* (Fig. 4C)*.*

#### Functional annotation

Unigenes obtained from the assemblies of two species were compared to four major databases (Nr, Swiss-Prot, KEGG, GO) for annotation purposes. Of the 289,093 unigenes of *S. calendulacea,* 150,846 (NR, 52.18%), 115,711 (Swiss-Prot, 40.03%), 60,116 (KEGG, 20.79%), and 97,957 (GO, 33.88%) were functionally annotated. By contrast, 119,350 (NR, 46.08%), 96,637 (Swiss-Prot, 37.31%), 49,159 (KEGG, 18.98%), and 83,712 (GO, 32.32%) of the 259,020 unigenes of *S. trilobata* were functionally annotated (Table 2).

**Table 2:** Functional annotation of unigene in *S. calendulacea* and *S. trilobata.*

Annotated-database	*S. calendulacea*		*S. trilobata*	
	Number	Percentage (%)	Number	Percentage (%)
Total unigenes	289,093	100	259,020	100
Nr-annotated	150,846	52.18	119,350	46.08
Swissprot-annotated	115,711	40.03	96,637	37.31
KEGG-annotated	60,116	20.79	49,159	18.98
GO-annotated	97,957	33.88	83,712	32.32

**Figure 5:**
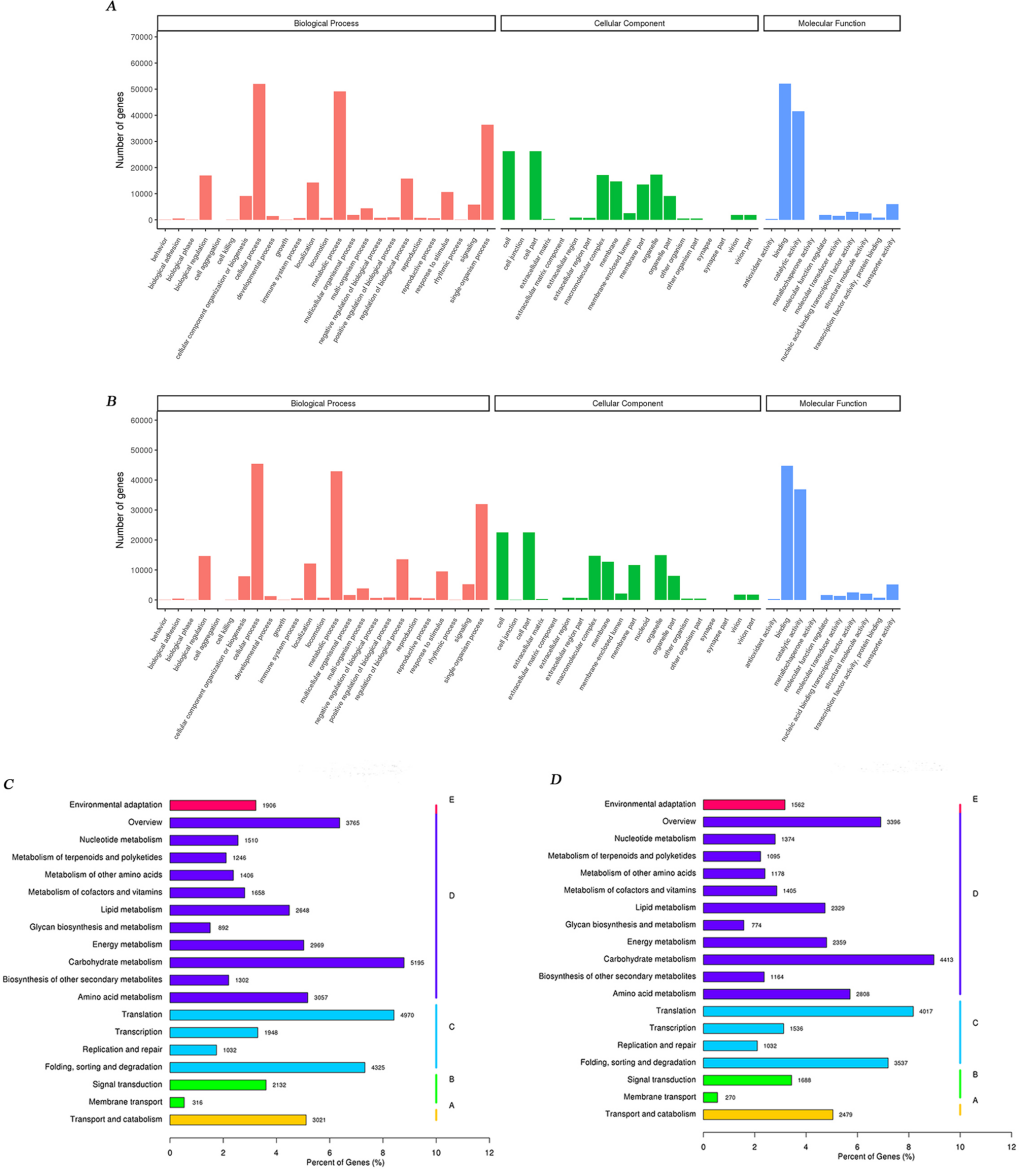
Gene ontology (GO) terms and Kyoto Encyclopedia of Genes and Genomes (KEGG) pathway annotations of unigenes by Blast2GO and KOBAS, respectively. The GO classifications of *S. calendulacea* (**A**) and *S. trilobata* (**B**)*,* included three major classification categories: Biological processes (Red), cellular components (Green) and molecular functions (Blue)*.* The x-axis represents different GO terms, and the number of genes is shown on the y-axis. The unigenes for *S. calendulacea* (**C**) and *S. trilobata* (**D**) were assigned to KEGG pathways. The x-axis represents the percentages of genes numbers in the pathways, while the y-axis represents the different pathways. Different bar colors represent different pathways.

Gene ontology (GO) terms were assigned to the annotated sequences from *S. calendulacea* and *S. trilobata* and were categorized into the three GO categories, namely, biological processes, molecular functions and cell components. The results showed that the distribution and percentages of the assigned gene functions were similar in both species (Fig. 5A,B). With regard to biological processes, *S. calendulacea* and *S. trilobata* were prominent in metabolic processes (50.95%/51.26%), cell processes (53.98%/54.23%) and single-organism processes (37.70%/38.14%), with more gene numbers. There were high proportions of gene numbers in the two species were involved in cell components, including cells (27.22%/26.93%) and cell parts (27.22%/26.93%). Similarly, genes with had high percentages of binding (53.99%/53.51%) and catalytic activity (43.06%/ 44.02%) were represented in the molecular function category.

In addition, KEGG analysis results indicated that there were 19 categories of unigenes in both species, such as environmental adaptation, nucleic acid metabolism and other entries (Fig. 5C,D). In these entries, *S. calendulacea* and *S. trilobata* had the highest proportion of genes in the carbon metabolism pathway and translation pathway, followed by amino acid biosynthesis, lipid metabolism and other biological processes.

#### Analysis of photosynthesis pathway and verification by real-time qPCR

**Figure 6:**
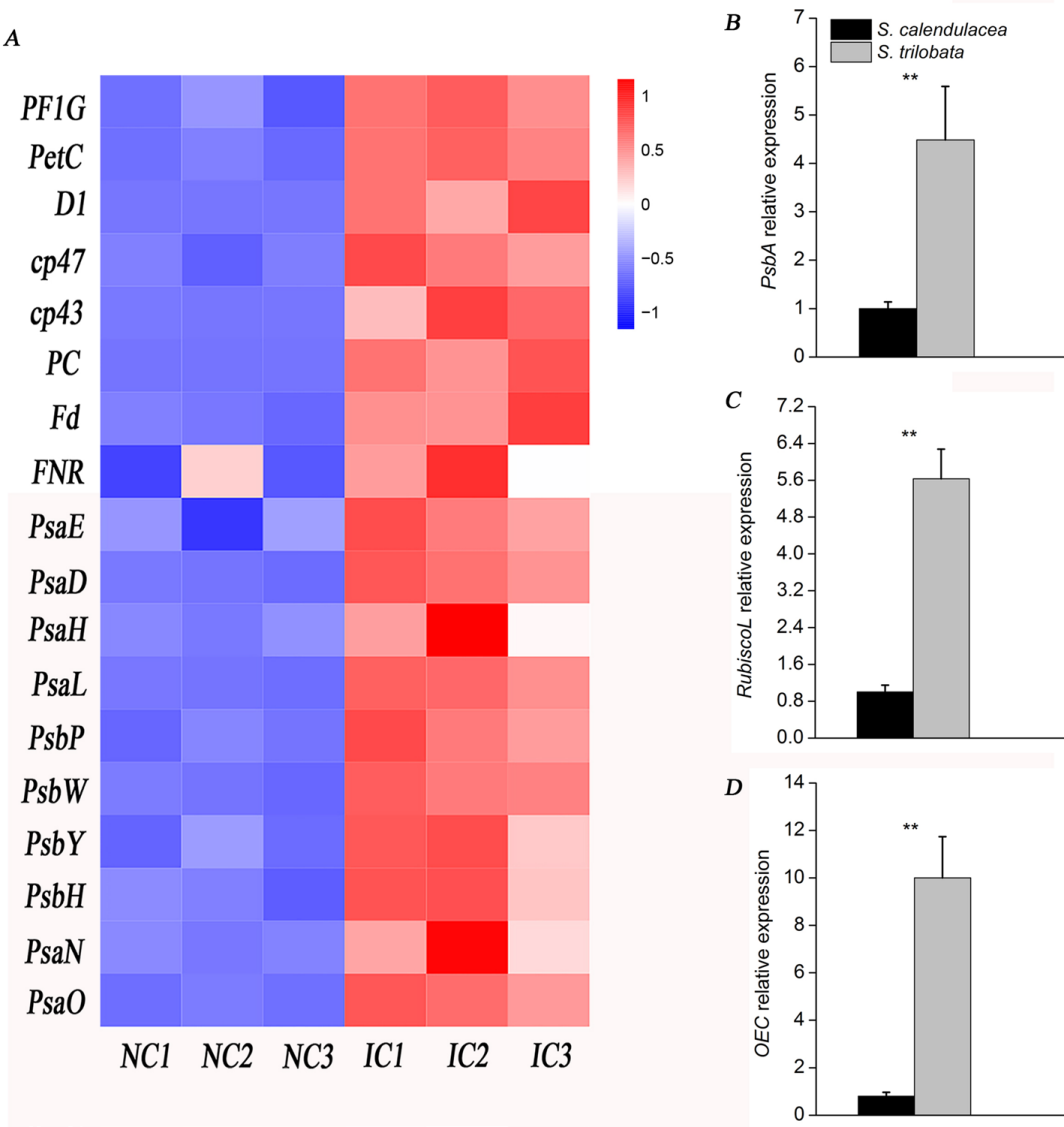
Comparison of the expression of photosynthetic genes in *S. calendulacea* (NC1–NC3) and *S. trilobata* (IC1–IC3) (**A**). Relative expression of the photosynthetic pathway, photosystem II D1 protein gene (*PsbA*) (**B**), Rubisco large subunit gene (*RubiscoL*) (**C**) and oxygen-evolving complex gene (*OEC*) (**D**) in the two species (n = 6). The glyceraldehyde 3-phosphate dehydrogenase (*GAPDH*) gene was used for normalization. The error bars represent the standard deviations (SDs), and the asterisks indicate significant differences according to two-sided Student’s t-test (*P < 0.05, **P < 0.01).

The photosynthetic pathway was further analyzed based on the previous results of photosynthetic physiology analysis and unigene functional annotation. In this study, we found that many photosynthetic genes, including *CP43, CP47* and *PsbA* in photosystem II, *PsaD* in photosystem I, *PetC* in cytochrome b6/f, the ferredoxin (Fd) gene (*PetF*) and ferredoxin-NADP^+^ reductase (FNR) gene (*PetH*) in photosynthethic electron transport, were higher expressed in leaves of *S. trilobata,* while lower in *S. chinensis* (Fig. 6A). The key protease genes of photosynthesis were also confirmed by qPCR. The results showed that the expression of the photosystem II D1 protein gene (*PsbA*) in *S. trilobata* was nearly 10 times higher than that in *S. chinensis* (Fig. 6B). In addition, the Rubisco large subunit (*RubiscoL*) and oxygen-evolving complex (OEC) genes (*PsbP*) were also highly expressed in *S. trilobata*, which were at 7 and 4.5 times higher than that of *S. calendulacea*, respectively. The qPCR results were consistent with those of RNA-Seq (Fig. 6C,D).

## Discussion

Photosynthesis is the sum of a series of complex metabolic reactions. It is the basis for the survival of the biological community and the material and energy basis for the growth and development of plants^26,45^. Photosynthetic capacity plays an important role in plant yield^27,28^. *S. trilobata* is one of the fast-growing invasive plants in South China. In the invaded area, *S. trilobata* has a large leaf area, leaf biomass and total biomass per plant were much higher than those of native species, *S. calendulacea* (Fig. 1A,D,E). This may be attributed to the fact that the photosynthetic capacity of *S. trilobata* is stronger than that of *S. calendulacea*. Thus, we analyzed the difference in the photosynthetic capacity of *S. trilobata* and its native plants by combined analysis of the physiological ecology and transcriptome. This could better explain the molecular mechanism underlying invasion by this species.

In this study, we found that the gas exchange parameters per unit leaf area of *S. trilobata* were higher than those of the native plant, including P_n_, G_s_, T_r_ and C_i_, which ensures that *S. trilobata* has higher photosynthetic capacity in the invasion area (Fig. 2A–D). At the same time, the changes in WUE also showed that *S. trilobat*a had higher light energy and water utilization efficiency than *S. calendulacea* (Fig. 2E). This result was consistent with the results of the field investigation of Wu et al. Studies have shown that Chl fluorescence is a probe of photosynthesis^26,29^. Compared with *S. calendulacea*, the ETR and Φ_PSII_ were higher in the leaves of *S. trilobata* (Fig. 2F,G), which indicated that the high photosynthetic capacity was related to its strong electron transfer ability^26^. It has been well acknowledged that Chl and Rubisco have often been considered indices of the light-harvesting and Calvin cycle capacities of leaves, respectively, which play an important role in photosynthesis capacity^30–32^. The leaves of *S. trilobata* had higher Chl and Rubisco contents than those of *S. calendulacea*, which was consistent with the trend observed for P_n_ (Fig. 3C,G). Rubisco/Chl had a higher ratio in *S. trilobata*, indicating that Rubisco accumulation was faster than Chl content (Fig. 3H). This could effectively prevent excess light energy from damaging the light system^30^. In contrast, the Chl *a/b* and Car/Chl ratio in *S. trilobata* were lower than those in the native species (Fig. 3E,F). The main reason may be that the synthesis of Chl *b*, the main photopigment, was faster than that of Chl *a* (Fig. 3A,B). This could ensure that *S. trilobata* leaves captured more light energy for photosynthesis and had higher photosynthetic efficiency. In contrast, the light system of *S. calendulacea* was more prone to oxidative stress than that of *S. trilobata* under the same conditions*.* It was found that the ROS (including O_2_^-^ and H_2_O_2_) accumulation in *S. chinensis* were much more severe than that in *S. trilobata* (Supplement Fig. 1A). ROS accumulation can directly or indirectly initiate membrane lipid peroxidation, resulting in membrane damage and electrolyte leakage^33^. The MDA content and relative conductivity in *S. chinensis* were higher than those of *S. trilobata* (Supplement Fig. 1B,C), indicating that the activities of SOD, POD and CAT in *S. chinensis* were also higher (Supplement Fig. 1D–F). Studies have shown that high antioxidant enzyme activity is an important defense mechanism for ROS scavenging^34^. In addition, we found that compared with *S. trilobata*, these was higher carotenoids content (Fig. 3D) and NPQ (Fig. 2H) in the leaves of *S. calendulacea*, which implied that the dissipation of excess light energy was also one of the protective mechanisms of photosynthetic organs in leaves^35^.

These physiological findings were further corroborated by RNA-Seq and real-time qRT-PCR assisted gene expression analysis (Fig. 6). The expression levels of genes in *S. trilobata* were different from those in *S. calendulacea* (Fig. 4C). By further comparing the differences of expression in the the photosynthetic pathway genes, we found that the genes encoding key proteases were highly expressed in the photosystem of *S. trilobata* (Fig. 6A). There are two photosystems, namely, photosystem II (PSII) and photosystem I (PSI), on the plant thylakoid membrane, which are responsible for capturing and converting light energy, respectively^36,46^. The D1 protein, a key protein subunit of PSII, is encoded by the *PsbA* gene. We found that *S. trilobata* had higher expression levels of the *PsbA* gene than the native plant (Fig. 6B). This may be due to the high efficiency of the D1 protein synthesis and turnover process, which ensures a high photosynthetic rate in chloroplasts^37^. Moreover, the gene encoding protein II (*PsbP*) of OEC was composed of three peptide chains and a manganese polymer with molecular weights of 16, 23 and 33 kDa, respectively. The expression level of the *PsbP* gene in *S. trilobata* was high (Fig. 6D), which indicated that PSII had strong stability with the manganese polymer and ensured its strong photosynthetic ability. This was consistent with the results showing that OEC expression in *Spiraea tomentosa* was decreased and adapted to a low-light environment with low photosynthetic capacity^38^. PSI, a multiprotein pigment complex, is involved in the coordination of the synthesis of Chl and carotene^39^. It was also found that the protease genes including *PsaE, PsaD* and *PsaO* were highly expressed in *S. trilobata*, which was consistent with the high content of Chl in the leaves. In addition, the protease complexes related to photosynthetic electron transfer, including *PetE*, *PetF* and *PetH*, were encoded by the *PC*, *FD* and *FNR* genes respectively, which indicated that the leaves of *S. trilobata* had higher photosynthetic electron transfer capacity. In our study, we also found that Rubisco, the cornerstone of photosynthesis, had high gene expression levels in *S. trilobata* (Fig. 6C). This ensured that the leaves of *S. trilobata* have high carbon assimilation capacity. Thus, the high expression of the key protein-encoding genes in the photosynthetic system was one of the important reasons for the high photosynthetic capacity of *S. trilobata*.

Therefore, *S. trilobata* not only has high net photosynthetic capacity per unit, but also has a larger leaf surface, which is mainly due to the regulation of two major hormones in the leaves, including CTK and auxin (Fig. 1B,C). Studies have found that phytohormones play important roles in leaf development^40^. They could cause an increase in cell division and cell growth in the leaves of *S. trilobata*. A large leaf surface could ensure that plants obtain more light energy for photosynthesis^41^.

In conclusion, strong photosynthetic capacity is one of the inherent advantages of *S. trilobata.* The key enzyme genes in the light system of *S. trilobata* were highly expressed and could regulate its high photosystem activity and ETR. This could ensure its strong light harvesting ability, and carbon assimilation ability, and high photosynthetic rate per unit leaf area. In addition, the leaf area was also large under the action of CTK and Auxin. These factors ensured that *S. trilobata* had higher plant biomass than the native plant, allowing successful invasion by *S. trilobata* in South China.

## Materials and methods

### Collection and cultivation of plants

*S. calendulacea* and *S. trilobata* were collected from the South China Botanical Garden, Guangzhou, China. The stems were cut into 8–10 cm lengths with two internodes for cultivation via cuttings in an incubator. The incubation conditions included a light intensity of 100 μmol m^−2^ s^−1^, a photoperiod of 14 h (10 h of darkness) and a temperature of 25 °C. After cultivation for half a month, the cutting seedlings were transplanted into pots containing Hoagland’s nutrient solution. After 30 days of cultivation, leaves of the two species with similar growth were collected for imaging with a digital camera (D7000, Nikon, Japan), and the leaf biomass and total biomass of individual plants were measured. The experiment was repeated five times.

### Determination of plant hormone content

Leaf samples of 0.05 g were weighed and add 1 mL of precooled PBS buffer to fully grind the samples. The grinding solution was poured into a 2 mL centrifuge tube, which was subsequently centrifuged at 4000 rpm, 10 min. Its supernatant was used for the determination of two plant hormones, including auxin and CTK. The determination method was referred to the instructions of the kit (Shenzhen Ziker Biological Technology Co., Ltd., Shenzhen, Guangdong, China). The kits were all tested by double antibody one-step sandwich enzyme-linked immunosorbent assay (ELISA). The absorbance (OD value) was measured at 450 nm wavelength with a multimode plate reader (EnSpire, PerkinElmer, USA), and the sample concentration was calculated.

### Determination of Chl fluorescence parameters

Chl fluorescence parameters of *S. trilobata* and *S. calendulacea* were measured using a Chl fluorescence imaging system (CF Imager, Technological Ltd. Colchester, UK). Five 8 mm leaf discs were placed in 12-well plates (each hole was filled with water). The leaf discs were adapted to the dark for 20 min before measurement. The determination procedure was set as follows: the saturated light pulse was 6162 mol m^−2^ s^−1^ (pulse time 1 s), the F_m_ was induced, and the photochemical light intensity was 800 mol m^−2^ s^−1^. The photosynthetic electron transfer efficiency (ETR), actual photochemical efficiency (Φ_PSII_) and non-photochemical quenching (NPQ) were measured as described by Li et al.^42^.

### Determination of Chl content

Fresh leaf samples of 0.05 g were weighed and placed in 10 mL centrifugal tubes. They were quickly crushed with metal rods, and then 4 mL of an 80% acetone solution was added and extracted overnight at 4 °C. The absorbance of the extract was determined at 663 nm and 645 nm using a UV–Vis 2450 spectrophotometer (Shimadzu, Tokyo, Japan), and 80% acetone was used as a blank control. The contents of Chl *a*, Chl *b* and Chl were calculated according to the methods of Wellburn^43^.

### Determination of gas exchange

Gas exchange parameters of leaves were measured by a LI-COR 6400 portable photosynthesis system (LI-COR, Lincoln, Nebraska, USA) on a sunny morning (8:30–11:30). The humidity was 55 ± 5%, and the leaf temperature was 30 °C. Photosynthesis in leaves was stabilized by light adaptation. The photon flux density (PPFD) was as the growth of light intensity of the plant, which was 800 μmol m^−2^ s^−1^. The net photosynthetic rate (P_n_), intercellular CO_2_ concentration (C_i_), stomatal conductance (G_s_), and transpiration rate (T_r_) of the leaves were recorded when the parameters of photosynthesis were stable.

### Determination of Rubisco protein content

Leaf samples weighing 0.05 g were placed into a mortar with a small amount of quartz sand and ground with 2 mL of plant extraction buffer (pH 7.8, including 50 mM Tris–HCl, 20 mM MgCl_2_, 1 mM EDTA-Na_2_, 10 mM mercaptoethanol, and 10 mM PMSF). The fluid obtained after grinding was centrifuged at 13,000 rpm, 4 °C for 10 min. The supernatant was mixed with an equal volume of 2 × protein loading buffer in a boiling water bath for 5 min and then stored at 4 °C. Rubisco protein was analyzed by sodium dodecyl sulfate polyacrylamide gel electrophoresis (SDS-PAGE), including 4% concentrated gel and 12.5% separated gel^44^. The amount of protein sample was 20 μL, the concentration voltage was 75 V for 30 min and the separation voltage was 110 V for 60 min. The large and small subunits (55 and 15 kDa, respectively) of the Rubisco protein were identified according to molecular mass, and the included marker was stained by Coomassie Brilliant Blue R-250. ImageJ software was used to analyze the gray value of the Rubisco protein band.

### Total RNA extraction

The total RNA from *S. calendulacea* and *S. trilobata* was extracted by TRIzol reagent (Promega). The RNA quality of the samples was determined by UV spectrophotometry and agarose gel electrophoresis; the OD_260/280_ and OD_260/230_ absorbance ratio were used to evaluate RNA purity, and RNA integrit was confirmed by 1% agarose gel electrophoresis. Samples with poor quality were re-extracted.

### Library construction and transcriptome sequencing

The Novogene NGS RNA Library Prep Kit was used to construct the libraries from the total RNA samples of the two species. The constructed libraries were tested for quality by an Agilent 2100 Bioanalyzer and ABI Step One Plus Real-time PCR System, and the libraries were paired-end sequenced on an Illumina HiSeq 4000 platform after passing the quality test. Sequence data were submitted to the NCBI Sequence Read Archive under accession numbers SRR8755022\SRR8755023\SRR8755024 (*S. calendulacea*) and SRR8755025\SRR8755020\SRR8755021 (*S. trilobata*).

### Sequencing data assembly

To obtain high-quality data (clean reads), raw reads were filtered to remove sequences with adapters and low-quality sequences. Clean reads were assembled using Trinity, and then sequence clustering was performed with CD-HIT; the remaining sequences were defined as unigenes.

### Functional annotation

To obtain the direction, function, and pathway annotations of unigenes, the unigenes of two species were searched against the nonredundant protein (Nr) database, Swiss-Prot protein database, and the Kyoto Encyclopedia of Genes and Genomes (KEGG) pathway database using BLASTx with an *E*-value cut-off of < 10^−5^. Gene ontology (GO) annotation of unigenes for describing biological processes, molecular functions and cellular components was performed using Blast2GO software.

### Detection of photosynthetic gene expression by real-time quantitative PCR

RNA was extracted from leaves with TRIzol reagent according to the manufacturer's instructions. The cDNA was synthesized by TopScript RT DryMIX (dT18) (Enzynomic, Daejeon, Korea) and the M-MLV reverse transcriptase kit (Takara). The relative expression of genes was determined by a Bio-Rad CFX96 Real-Time PCR System (CFX96, Bio-Rad, USA). The SYBR Premix Ex Taq II Kit (Takara) was used to analyze the relative expression of the oxygen-evolving complex-coding gene (*OEC*), D_1_ protein-coding gene (*PsbA*) and Ribose-1,5-diphosphate carboxylase/oxygenase gene (*RubiscoL*) in PSII. Each 10 μL aliquot of reaction solution contained: 5 μL of SYBR Premix Ex Tap II, 0.2 μL of each primer, template cDNA (< 100 ng) and added ddH_2_O in a total volume of 10 μL. The reaction cycle was as follows: 95 °C for 30 s, followed by 39 cycles (95 °C for 5 s, 60 °C for 34 s), and 1 cycle for recording a melt curve at 95 °C for 10 s and 60 °C for 5 s. The expression of related genes was calculated by 2^−△△CT^. Glyceraldehyde-3-phosphate dehydrogenase (GAPDH) was used as an internal reference. The forward and reverse primers corresponding to the internal reference gene and the target gene are shown in Table 3.

**Table 3:** Primer design of internal and target genes.

Gene name	Forward primer (5′–3′)	Reverse primer (5′–3′)
*GAPDH*	CTGCTTCATTCAACATC	CTCACGGTCAGATCAACA
*OEC*	TGCAGCAAGGGATAAGGATGT	ACAAATGAAAGAGCATGAACAAAGA
*PsbA*	TGGAGGAGCAGCAATGA	GCGAAAGCGAAAGCCTA
*Rubisco L*	CGGTCTCTCCAGCGCATAAA	CGCCTCACGGTATCCAAGTT

### Statistical analysis

Excel 2016 was used for data collection and statistical analysis, SPSS19.0 (IBM SPSS, Chicago, USA) was used for one-way ANOVA and the LSD test, and OriginPro 8.0 (OriginLab, Northampton, MA, USA) and Adobe Photoshop CC 2014 (Adobe Systems Inc., USA) were used for preparing figures.

## Supplementary information

Supplementary Information.

Supplementary Figure.
