# Supplementary material for: Comparative physiological and transcriptomic analyses of photosynthesis in Sphagneticola calendulacea (L.) Pruski and Sphagneticola trilobata (L.) Pruski
Source: Sci Rep. 2020 Oct 20;10:17810. doi: 10.1038/s41598-020-74289-1 (PMC7576218; doi:10.1038/s41598-020-74289-1)
Supplement: Supplementary file 1 — Supplementary Information. [file 41598_2020_74289_MOESM1_ESM.docx]

**Comparative physiological and transcriptomic analyses of photosynthesis in *Sphagneticola calendulacea* (L.) Pruski and *Sphagneticola trilobata* (L.) Pruski**

Min-ling Cai^1†^, Qi-lei Zhang^1†^, Jun-jie Zhang^1^, Wen-qiao Ding^1^,

Hong-ying Huang^2*^,  Chang-lian Peng^1*^

^1^ Guangzhou Key Laboratory of Subtropical Biodiversity and Biomonitoring, Guangdong Provincial Key Laboratory of Biotechnology for Plant Development, College of Life Sciences, South China Normal University, Guangzhou 510631, PR China.

^2^ College of Chemistry & Biology and Environmental Engineering, Xiangnan University, Chenzhou, Hunan, 423043, PR China

^†^ These authors contributed equally to this work.

^*^ Correspondence: [pengchl@scib.ac.cn](mailto:pengchl@scib.ac.cn) (Chan-lian Peng), [xnxyhhy@xnu.edu.cn](mailto:xnxyhhy@xnu.edu.cn) (Hong-ying Huang)

**Supplementary**

**Materials and methods**

**Determination of antioxidant enzyme activity**

A total of 0.1 g of leaves from the two *Sphagneticola* species was homogenized in a mortar with 2 mL of extraction buffer containing 50 mM potassium phosphate buffer (pH 7.8), 100 mM ethylenediamine-tetraacetic acid (EDTA), 0.1% (w/v) Triton X-100 and 2% (w/v) insoluble polyvinylpyrrolidone (PVP). The homogenate was centrifuged for 10 min at 12 000 × g and 4 °C. The supernatant was used for enzyme activity assays. Enzymatic antioxidants including SOD, POD and CAT were measured by the modified method described by Tan et al. (2008), Du et al (2009), Jiang et al. (2001), and Nakano and Asada (1981)^1-4^, respectively. SOD activity was measured using the reaction mixture containing 50 mM phosphate buffer (pH 7.8), 20 μM riboflavin, 130 mM methionine, 0.1 μM EDTA, 750 mM NBT and 100 μL of enzyme extract and the absorbance at 560 nm was recorded. The POD reaction solution was composed of 50 mM potassium phosphate buffer (pH 7.0), 30 mM H_2_O_2_ and guaiacol. The reaction was initiated by adding 100 μL of enzyme extract, and the increase in absorbance at 470 nm for 3 min was recorded. The decline in absorbance of CAT activity was observed for 2 min at 240 nm. The assay solution was prepared by mixing 30 mM H_2_O_2_ and 100 μL of enzyme extract.

**Determination of relative membrane permeability and malondialdehyde content**

Relative membrane permeability was estimated as described by Zheng et al (2019)^5^. The conductivity was recorded by a DDS-11C conductometer (Shanghai Dapu Instruments). Three leaf discs (8 mm in diameter) were immersed in 5 mL of double-distilled water for 3 h at room temperature, and the conductivity was recorded as R1. After the solution was heated in a boiling water bath for 40 min, the conductivity was measured an recorded as R2. The cell membrane leakage rate was calculated using the formula: (R1/R2)× 100%.

Malondialdehyde (MDA) content was determined using the thiobarbituric acid (TBA) method. Leaves (0.1 g) were crushed into homogenate with 2 mL of 0.1% (w/v) trichloroacetic acid (TCA) solution. The homogenate was centrifuged at 13,000 rpm for 10 min. The reaction mixture (2 mL) contained 1 mL of supernatant and 1 mL of 0.6% (w/v) TBA. It was cooled quickly on ice after incubation in boiling water for 30 min. The absorbance of the mixture was measured at wavelengths of 600, 532 and 450 nm wavelengths. The MDA content was calculated according to the formula of Wang and Jin (2005)^6^.

**Superoxide** (O_2_^-^) **and hydrogen peroxide (H_2_O_2_) detection by histochemical staining**

Superoxide (O_2_^-^) and hydrogen peroxide (H_2_O_2_), two of the most important ROS, were detected by using 3,3ʹ-diaminobenzidine (DAB) and nitrotetrazolium blue chloride (NBT) staining, respectively^7^. Fresh leaves were infiltrated with phosphate buffer (50 mM, pH 7.0) containing 0.5 mg mL^−1^ DAB under a vacuum for 30 min. Subsequently, the leaves were incubated in the dark for 8 h. H_2_O_2_ accumulation in leaves could be observed as brown spots after DAB staining. However, O_2_^-^ accumulation in leaves could be observed as blue spots after incubation for 2 h. The leaves were infiltrated with phosphate buffer (50 mM, pH 6.4) containing 0.1% (w/v) NBT and 10 mM sodium azide (NaN_3_) under a vacuum for 30 min. After staining, 95% (v/v) ethanol was used to bleach Chl in the leaves.

**Results**

In this study, the accumulation of reactive oxygen species (ROS) in the leaves of two *Sphagneticola* species was compared. It was found that the ROS (including O_2_ ^-^ and H_2_O_2_) accumulation in the leaves of *S. trilobata* was lower than that of native *S. chinensis*, and the accumulation of O_2_ ^-^ was the highest in the leaves of *S. chinensis* (**Supplement Figure 1A**). We further determined that the MDA content and relative conductivity of the leaves of *S. trilobata* were significantly lower than those of native *S. chinensis* (*p* < 0.05), decreasing by 17.5% and 27.9% respectively (**Supplement Figure 1B-C**). In addition, antioxidant enzymes are important defense systems of the ROS scavenging system. The results showed that compared with native species, CAT activity in *S. trilobata* leaves maintained a low level, but there was no significant difference between the species (**Supplement Figure 1F**). However, the activities of SOD and POD in *S. trilobata* were significantly lower than those in *S. chinensis* (*p* < 0.05), and their reduction rates were 83.3% and 43.2%, respectively (**Supplement Figure 1D-E**).

Reference:

1. Tan, W., Liu, J., Dai, T., [Cao](https://link.springer.com/article/10.1007/javascript:;), W., & Jiang, D. Alterations in photosynthesis and antioxidant enzyme activity in winter wheat subjected to post-anthesis water-logging. *Photosynthetica*. **46**, 21e27 (2008). doi: 10.1007/s11099-008-0005-0.
2. Du, H., Wang, Z., & Huang, B. Differential responses of warm-season and cool season turfgrass species to heat stress associated with antioxidant enzyme activity. *J Am Soc Hortic Sci*. **134**, 417–422 (2008). doi: 10.21273/JASHS.134.4.417.
3. Jiang, Y., & Huang, B. Drought and heat stress injury to two cool-season turfgrasses in relation to antioxidant metabolism and lipid peroxidation. *Crop Sc*i. **41**, 436–442 (2001). doi: 10.2135/cropsci2001.412436x
4. Nakano, Y., & Asada, K. Hydrogen peroxide is scavenged by ascorbate-specific peroxidase in spinach chloroplasts. *Plant Cell Physiol*. **22**, 867–880 (1981). doi: 10.1093/oxfordjournals.pcp.a076232
5. Zheng, X, T., Chen, Y, L, Zhang, X, H., Cai, M, L., Yu, Z, C., & Peng, C, L. ANS-deficient Arabidopsis is sensitive to high light due to impaired anthocyanin photoprotection. *Funct Plant Biol* (2019). doi:10.1071/fp19042.
6. Wang, H., & Jin, J, Y. Photosynthetic rate, chlorophyll fluorescence parameters, and lipid peroxidation of maize leaves as affected by zinc deficiency. *Photosynthetica* **43**, 591–596 (2005). doi: 10.1007/s11099-005-0092-0.
7. Cai, M, L. *et al.* Photosynthetic compensation of non-leaf organ stems of the invasive species *Sphagneticola trilobata* (L.) Pruski at low temperature. *Photosynth Res* (2020). doi: 10.1007/s11120-020-00748-5.
