# Supplementary figures and images for: Comparative physiological and transcriptomic analyses of photosynthesis in Sphagneticola calendulacea (L.) Pruski and Sphagneticola trilobata (L.) Pruski
Source: Sci Rep. 2020 Oct 20;10:17810. doi: 10.1038/s41598-020-74289-1 (PMC7576218; doi:10.1038/s41598-020-74289-1)

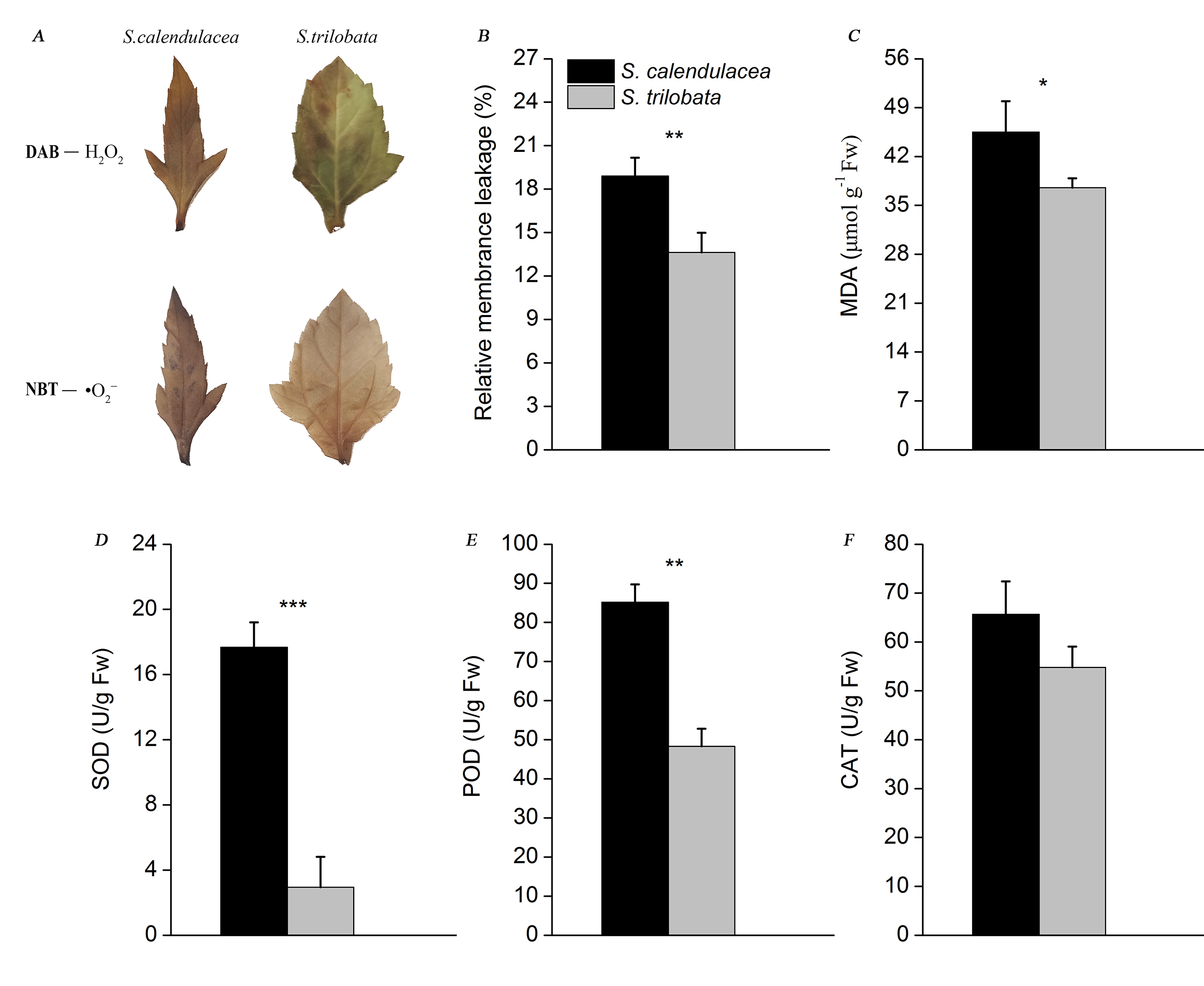

Supplement: Supplementary file 2 — Supplementary Figure. [file 41598_2020_74289_MOESM2_ESM.jpg]
